# Effect of Tofogliflozin on Body Composition and Glycemic Control in Japanese Subjects with Type 2 Diabetes Mellitus

**DOI:** 10.1155/2018/6470137

**Published:** 2018-01-08

**Authors:** Shinji Kamei, Masahiro Iwamoto, Miyuki Kameyama, Masashi Shimoda, Tomoe Kinoshita, Atsushi Obata, Tomohiko Kimura, Hidenori Hirukawa, Fuminori Tatsumi, Kenji Kohara, Shuhei Nakanishi, Tomoatsu Mune, Kohei Kaku, Hideaki Kaneto

**Affiliations:** ^1^Department of Diabetes, Endocrinology and Metabolism, Kawasaki Medical School, Kurashiki, Japan; ^2^Iwamoto Internal Medicine Clinic, Kagawa, Japan

## Abstract

Sodium-glucose cotransporter 2 inhibitor tofogliflozin is a new type of antidiabetic drug for individuals with type 2 diabetes mellitus (T2DM). The aim of this study was to examine in which type of individuals and/or under which conditions tofogliflozin could exert more beneficial effects on body composition and/or glycemic control in Japanese individuals with T2DM. We retrospectively evaluated the effects of tofogliflozin on body composition and/or glycemic control in individuals with T2DM who newly started taking tofogliflozin. After tofogliflozin treatment, body weight was significantly reduced and HbA1c levels were significantly decreased. Body fat mass, skeletal muscle mass, and skeletal muscle index, a marker for sarcopenia, were also reduced after the treatment. In univariate analyses, there was a statistically significant association between the decrease of HbA1c level after tofogliflozin treatment (Δ HbA1c) and the following parameters such as HbA1c levels at baseline, visceral fat area (VFA) at baseline, and reduction of VFA after the treatment (Δ VFA). Furthermore, in multivariate analyses, HbA1c levels at baseline and duration of diabetes were independently associated with Δ HbA1c. These results suggest that tofogliflozin would be more suitable for relatively obese individuals whose duration of diabetes is relatively short.

## 1. Introduction

SGLT2 inhibitors improved glycemic control by reducing filtered renal glucose reabsorption from the proximal tubule and promoting excretion of excess glucose [1–5]. SGLT2 inhibitors are promising drugs in view of reduction of glucose toxicity which is often observed in T2DM. Tofogliflozin is a highly selective SGLT2 inhibitor. It has been shown that tofogliflozin, at a dose of 20 mg orally once daily, is well tolerated, without increased risk of hypoglycemia in phase 2 and 3 studies [2, 5].

Approximately 180 g/day of glucose is filtered by the kidneys every day, but about 90% of such glucose is reabsorbed by SGLT2 in the proximal renal tubule [6–9]. It has been shown that SGLT2 inhibitors reduce glucose reabsorption in the kidney and increase urinary glucose excretion, which leads a substantial additional calorie loss. It has been well established that SGLT2 inhibitors have effects on weight reduction due to calorie loss [10]. Therefore, such effect is one of the ideal benefits of SGLT inhibitors within the framework of diabetes treatment. It remains unknown, however, how SGLT inhibitors change body composition.

Dual-energy X-ray absorptiometry (DEXA) is a gold standard method for evaluation of body composition, but it has several problems such as a high price, an exposure to ionising radiation and a lack of portability [11]. Recently, bioelectric impedance assessments (BIA) are often used to characterize body composition, because of its simplicity and portability [12–15]. The BIA method is based on the phenomena that the conduction of electrical current through body fluids is much greater in fat-free mass compared to fat mass, because fat-free mass contains all body fluids and electrolytes [16]. Thus, electrical resistance and reactance together with body weight and height can reliably estimate body composition [17–19]. There are few data, however, about their changes during treatment of SGLT inhibitors.

The aim of this study is to examine in which type of individuals and/or under which conditions tofogliflozin exerts more beneficial effects on body composition and/or glycemic control in Japanese individuals with T2DM.

## 2. Methods

### 2.1. Study Design

We retrospectively examined a total of 37 subjects with T2DM who visited Iwamoto Internal Medicine Clinic or our hospital between July 2014 and March 2015. The subjects who had taken other SGLT2 inhibitors during 6 months preceding the study were excluded. Tofogliflozin (20 mg/day) was orally administered. As generally recommended for SGLT2 inhibitors, we encouraged the subjects to drink approximately 1000 ml of additional water to avoid several potential adverse events such as dehydration and urinary tract infection. We measured common clinical parameters, including height, weight, body mass index (BMI) (kg/m^2^), waist circumference (measured at the level of the umbilicus in the standing position), and blood pressure. We also examined the habitual lifestyle, including smoking, drinking, exercise, and diet. All of the patients were treated by caloric diet restriction. At every visit, after overnight fasting, blood samples were obtained from all patients for measuring the multiple metabolic parameters. Other common parameters associated with T2DM, including complete blood cells, blood chemistry, and diabetic and lipid profile, were also examined. The change in HbA1c level from baseline until the end of the follow-up period and the incidence of adverse events, including hypoglycemia and percentage of dropout due to adverse events, especially dehydration, urinary tract infection, and skin eruption, were also evaluated. The hospital ethics committee approved the study protocol (number 2552), and informed consent was obtained from each person by showing it in our homepage after a full explanation of the study. In Japan, this is a usual method for a retrospective study and this process per se was also formally approved by the hospital ethics committee.

### 2.2. Evaluation of Body Composition Parameters

Body composition variables were measured by a bioelectrical impedance body composition analyzer InBody 770 (InBody Japan Inc., Japan). Measurements were performed with the participants barefoot and in minimal clothing without any metallic materials. Body weight, visceral fat area (VFA), total body water, intracellular and extracellular water body fat mass, percent body of fat, soft lean mass, and skeletal muscle mass were measured before and after treatment with tofogliflozin. The appendicular skeletal muscle mass index (skeletal muscle mass/height (m)^2^) was calculated according to the method suggested by Janssen et al. [20]. Furthermore, we adopted the Asian criteria to evaluate the presence of sarcopenia [21].

### 2.3. Statistical Analysis

All data were analyzed using JMP version 11.0 and are presented as mean ± standard error (SE) or number (percentage). Because the distributions of serum triglyceride levels, U-Alb excretion, and HOMA (homeostasis model assessment) values were skewed, natural logarithmic transformation was applied in the statistical analysis. Changes in parameters from the baseline values in the same patient were evaluated using two-tailed paired *t*-test. Unpaired *t*-test was used to compare the differences in clinical characteristics between groups at baseline and after treatment, and the chi-square test was used for frequency distributions. Furthermore, a linear regression analysis was used to identify independent factors to predict the effects of tofogliflozin. *p* value < 0.05 was considered to be statistically significant.

## 3. Results

### 3.1. Clinical Characteristics of This Study Subjects

As shown in Table 1, clinical characteristics of this study subjects were as follows: age, 52.7 ± 1.9 years old; number (male/female), 37 (24/13); duration of diabetes, 11.3 ± 1.2 years; body weight, 89.0 ± 3.1 kg; BMI, 32.2 ± 0.8 kg/m^2^; HbA1c, 8.3 ± 0.2%; systolic blood pressure, 131.2 ± 2.6 mmHg; diastolic blood pressure, 76.2 ± 1.9 mmHg; triglyceride, 216.3 ± 24.4 mg/dL; LDL cholesterol, 112.0 ± 4.4 mg/dL; HDL cholesterol, 48.3 ± 2.6 mg/dL; uric acid, 5.6 ± 0.3 mg/dL; and hematocrit, 45.6 ± 1.2% (mean ± SE). Number (percentage) of subjects with diabetic microangiopathy was as follows: peripheral neuropathy 9 (24.3%), retinopathy 10 (27.0%), and nephropathy; stage 1: 23 (62.2%), stage 2: 8 (21.6%), and stage 3: (16.2%), respectively. Number (percentage) of subjects with history of myocardial infarction and/or stroke was 3 (8.1%). Number (percentage) of concomitant medication was as follows: biguanide 25 (67.6%), DPP-IV inhibitor 21 (56.8%), sulfonylurea 19 (51.4%), and thiazolidine 3 (8.1%). Insulin user was 23 (62.2%) with total insulin dose of 52.9 ± 4.8 units (0.61 U/kg).

### 3.2. Effects of Tofogliflozin on Various Metabolic Parameters

As shown in Table 1, at 4 and 12 weeks after tofogliflozin treatment, body weight and BMI were significantly reduced compared to those before the treatment. Body weight 4 and 12 weeks after tofogliflozin treatment were 87.2 ± 3.1 kg and 85.7 ± 3.0 kg, respectively, both of which were significantly lower compared to baseline body weight (*p* < 0.01 and *p* < 0.01). Reduction from baseline body weight was −1.8 kg and −3.3 kg, respectively. HbA1c was also significantly decreased. HbA1c levels 4 and 12 weeks after tofogliflozin treatment were 7.7 ± 0.1% and 7.3 ± 0.1%, respectively, both of which were significantly lower compared to baseline HbA1c (*p* < 0.01 and *p* < 0.01). Reduction from baseline HbA1c was −0.6% and −1.0%, respectively, and at week 12, 78% of subjects reached HbA1c < 7% with tofogliflozin.

Systolic blood pressure was slightly decreased to 126.4 ± 2.5 mmHg at 4 weeks and 126.8 ± 2.4 mmHg at 12 weeks, but it did not reach a statistical significance. There was no difference in diastolic blood pressure before and after tofogliflozin treatment (Table 1). After the initiation of tofogliflozin treatment, LDL-C levels were decreased to 107.1 ± 4.8 mg/dL at 4 weeks and 107.2 ± 4.3 mg/dL at 12 weeks, but it did not reach a statistical significance. HDL-C levels 4 and 12 weeks after tofogliflozin treatment were 42.4 ± 2.0 mg/dL and 45.5 ± 2.0 mg/dL, respectively. There was no statistical significance between before and after treatment. At the same checkpoints, triglyceride levels were decreased to 214.1 ± 26.6 mg/dL and 173.2 ± 16.1 mg/dL, respectively, but it did not reach a statistical significance. After the initiation of tofogliflozin treatment, uric acid level was significantly decreased to 5.3 ± 0.2 mg/dL at 4 weeks and 4.9 ± 0.2 mg/dL at 12 weeks, both of which were significantly lower compared to baseline uric acid (*p* < 0.05 and *p* < 0.05) (Table 1).

During 24 weeks of treatment, 2 (5.3%) male patients experienced bacterial balanoposthitis, which is a known side effect of SGLT2 inhibitors, but these were transient and recovered soon. There was no change in hematocrit before and after tofogliflozin treatment (Table 1). Severe hypoglycemia was not observed, and hypotension and cerebral and coronary events associated with dehydration were not observed.

### 3.3. Evaluation of Tofogliflozin Effects on Body Composition Using a Bioelectrical Impedance Body Composition Analyzer InBody

To evaluate the effects of tofogliflozin on body composition, we evaluated it by using InBody.  Table 2 shows the changes of body composition evaluation using InBody 770. There was a significant difference in various markers between before and 4 or 12 weeks after the treatment. Body fat mass was significantly decreased after 4 and 12 weeks of tofogliflozin treatment (33.7 ± 1.7 kg at baseline; 32.3 ± 1.9 kg at 4 weeks, *p* < 0.01; 31.8 ± 1.8 at 12 weeks, *p* < 0.01). Reduction from baseline body fat mass was −1.4 kg and −1.9 kg, respectively. Visceral fat area (VFA) was also significantly decreased from baseline (157.7 ± 8.0 cm^2^ at baseline; 152.1 ± 9.0 cm^2^ at 4 weeks, *p* < 0.01; 150.5 ± 8.7 cm^2^ at 12 weeks, *p* < 0.01, resp.). Skeletal muscle mass in the whole body and in extremities was also significantly reduced from baseline (*p* < 0.01). Reduction from baseline skeletal muscle mass was −0.5 kg and −0.8 kg, respectively. Therefore, although skeletal muscle mass was reduced, the extent of reduction in the skeletal muscle was much smaller compared to that in body fat mass (−1.4 kg and −1.9 kg). Skeletal muscle index also showed statistically significant reduction; in male subject, 9.1 ± 0.2 kg/m^2^ at baseline; 8.9 ± 0.2 kg/m^2^ at 12 weeks, *p* < 0.01 and in female subjects, 7.0 ± 0.3 kg/m^2^ at baseline; 6.9 ± 0.3 kg/m^2^ at 12 weeks, *p* < 0.01. However, even after such reduction, skeletal muscle index was high enough and far from the cutoff values of the Asian sarcopenia criteria (male: <6.9, female: <5.5). Soft lean mass was also decreased after tofogliflozin treatment (*p* < 0.01). Total body water and intracellular and extracellular water were also significantly decreased especially during 4 weeks, suggesting that weight reduction for 0–4 weeks from baseline was mainly dependent on water loss.

### 3.4. Evaluation about Which Factor Determines the Effects of Tofogliflozin on Glycemic Control

To examine which factors are associated with the effect of tofogliflozin, we performed univariate analyses. As shown in Table 3, Δ HbA1c (4 weeks) and Δ HbA1c (12 weeks) were significantly associated with baseline HbA1c (*p* < 0.01 and *p* < 0.01). Δ HbA1c (4 weeks) and Δ HbA1c (12 weeks) were also significantly associated with baseline VFA (*p* < 0.01 and *p* < 0.01). In addition, Δ HbA1c (4 weeks) was significantly associated with Δ VFA (4 weeks) (*p* < 0.05), whereas Δ HbA1c (12 weeks) was not significantly associated with Δ VFA (12 weeks). Δ VFA (4 weeks) was significantly associated with baseline VFA (*p* < 0.01), whereas Δ VFA (12 weeks) was not significantly associated with baseline VFA.

Next, to examine which factor independently determines the effect of tofogliflozin, we performed multivariate analyses. As shown in Table 4, when we used baseline HbA1c, baseline VFA, and age as explanatory variables and Δ HbA1c (4 weeks) as an objective variable, baseline HbA1c was independently associated with Δ HbA1c (4 weeks) (model 1). When we used baseline HbA1c, baseline VFA, and duration of diabetes as explanatory variables and Δ HbA1c (4 weeks) as an objective variable, baseline HbA1c and duration were independently associated with Δ HbA1c (4 weeks) (model 2). Similarly, when we used baseline HbA1c, baseline VFA, and age as explanatory variables and Δ HbA1c (12 weeks) as an objective variable, baseline HbA1c was independently associated with Δ HbA1c (12 weeks) (model 3). When we used baseline HbA1c, baseline VFA, and duration of diabetes as explanatory variables and Δ HbA1c (12 weeks) as an objective variable, baseline HbA1c and duration were independently associated with Δ HbA1c (12 weeks) (model 4).

## 4. Discussion

In this study, we showed that tofogliflozin, an SGLT2 inhibitor, exerted beneficial effects on metabolic parameters such as body weight, HbA1c, and uric acid without severe adverse effects (Table 1). In addition, we evaluated the effect of tofogliflozin on body composition using InBody 770 and showed that body fat mass was substantially decreased accompanied by the reduction of body water as well as skeletal muscle mass (Table 2). Finally, we showed that tofogliflozin exerted larger effects on glycemic control in subjects with higher HbA1c levels or larger VFA at baseline and/or shorter duration of diabetes (Tables 3 and 4). These results suggest that tofogliflozin would be more suitable for relatively obese subjects whose duration of diabetes are relatively short.

As shown in Table 1, HbA1c level was significantly reduced after tofogliflozin treatment, but the reduction in HbA1c in this study was smaller compared to those which were previously reported in phase 2 and 3 studies [2, 5]. Reduction of body weight in this study was similar to those in the 2 and 3 study [2, 5]. Contrary to our expectations, blood pressure was not significantly reduced after tofogliflozin treatment and lipid profile showed no significant improvement in this study. In our study, although body weight was reduced, the subjects were still obese even after tofogliflozin treatment (BMI 31.3 ± 0.8 kg/m^2^), which is one of the possible reasons why blood pressure and lipid profile were not significantly improved in our case.

As shown in Table 2, body fat mass was more substantially decreased compared to the alteration of skeletal muscle mass; reduction of body fat mass at 4 and 12 weeks was −1.4 kg and −1.9 kg, respectively, and reduction of skeletal muscle mass at 4 and 12 weeks was −0.5 kg and −0.8 kg, respectively. In addition, although the skeletal muscle was significantly decreased, skeletal muscle index after such reduction was high enough and far from the cutoff values of the Asian sarcopenia criteria [21]. Therefore, we think that we do not need to be afraid of sarcopenia as far as we use SGLT2 inhibitors in an appropriate way.

In addition, total body water was also significantly decreased, although we encouraged the subjects to drink approximately 1000 ml of additional water to avoid several potential adverse events such as dehydration and urinary tract infection. Therefore, we think that some body weight reduction was dependent on water loss. Since intracellular and extracellular water was equally reduced, reduction of both water might have been related with the reduction of body weight.

It was reported that an 8-week administration of tofogliflozin improved glycemic control and reduced body weight and fat mass in Japanese subjects with T2DM (*n* = 17) [22]. Our study was performed with a bit larger number (*n* = 37) and longer observation period (3 months), but similar data were obtained. In addition, a 16-week double-blind study showed that tofogliflozin exerted beneficial effects in subjects with T2DM whose HbA1c levels were poorly controlled with insulin monotherapy or insulin plus a DPP-4 inhibitor [23]. These findings suggest that tofogliflozin is useful for good glycemic control and reduction of fat mass in a variety of situations.

It was reported that a 3-month treatment with ipragliflozin, another SGLT2 inhibitor, improved glycemic control and reduced fat mass in Japanese subjects with T2DM [24]. Since the data in our study with tofogliflozin were similar to those with ipragliflozin, we assume that the data in our study could be true for other SGLT2 inhibitors. While various kinds of SGLT2 inhibitors are available in clinical medicine especially in Japan, it would be important to know the difference in characteristics among various kinds of SGLT2 inhibitors. In order to directly compare such beneficial and/or adverse effects among various kinds of SGLT2 inhibitors, it would be necessary to perform further evaluation in the future.

There is a limitation in this study. Since this study was retrospective, unblinded, and uncontrolled and our sample size was small, a large-scale and randomized prospective study would be necessary to achieve a higher level of evidence. Since the subjects in this study were middle-aged and relatively obese, the data in this study are not necessarily true for aged and lean subjects.

Taken together, tofogliflozin exerts beneficial effects on metabolic parameters such as body weight, body mass composition, and HbA1c without severe adverse effects in middle-aged and relatively obese subjects with T2DM. In addition, tofogliflozin exerts larger effects on glycemic control in subjects with higher HbA1c levels or larger VFA at baseline and/or shorter duration of diabetes. These results suggest that tofogliflozin would be more suitable for relatively obese subjects whose duration of diabetes is relatively short.

## Figures and Tables

**Table 1:** Efficacy of tofogliflozin on various metabolic parameters.

Parameters	Baseline	4 weeks	*p*	12 weeks	*p*
Body weight (kg)	89.0 ± 3.1	87.2 ± 3.1	<0.01	85.7 ± 3.0	<0.01
Body mass index (kg/m^2^)	32.2 ± 0.8	31.5 ± 0.9	<0.01	31.3 ± 0.8	<0.01
HbA1c (%)	8.3 ± 0.2	7.7 ± 0.1	<0.01	7.3 ± 0.1	<0.01
Systolic BP (mmHg)	131.0 ± 2.6	126.4 ± 2.5	n.s.	126.8 ± 2.4	n.s.
Diastolic BP (mmHg)	76.3 ± 1.9	76.2 ± 1.9	n.s.	76.3 ± 1.7	n.s.
Triglyceride (mg/dL)	216.3 ± 24.7	214.1 ± 26.6	n.s.	173.2 ± 16.1	n.s.
LDL cholesterol (mg/dL)	112.0 ± 4.4	107.1 ± 4.8	n.s.	107.2 ± 4.3	n.s.
HDL cholesterol (mg/dL)	48.3 ± 2.6	42.4 ± 2.0	n.s.	45.5 ± 2.0	n.s.
Uric acid (mg/dL)	5.6 ± 0.3	5.3 ± 0.2	<0.05	4.9 ± 0.2	<0.05
Hematocrit (%)	45.6 ± 1.2	44.2 ± 1.2	n.s.	46.2 ± 0.7	n.s.

**Table 2:** Evaluation about the efficacy of tofogliflozin on body composition using InBody.

Parameters	Baseline	4 weeks	*p*	12 weeks	*p*
Body fat mass (kg)	33.7 ± 1.7	32.3 ± 1.9 (−1.4)	<0.01	31.8 ± 1.8 (−2.9)	<0.01
Skeletal muscle mass (kg)	29.8 ± 1.2	29.3 ± 1.2 (−0.5)	<0.05	29.0 ± 1.1 (−0.8)	<0.01
Skeletal muscle mass of extremities (kg)	M: 25.9 ± 0.8 F: 17.1 ± 0.8	25.5 ± 0.9 17.1 ± 0.8	<0.01 n.s.	25.2 ± 0.8 16.5 ± 0.9	<0.01 <0.01
Skeletal muscle index (kg/m^2^)	M: 9.1 ± 0.2 F: 7.0 ± 0.3	8.9 ± 0.2 7.0 ± 0.3	<0.01 n.s.	8.9 ± 0.2 6.9 ± 0.3	<0.01 <0.01
Soft lean mass (kg)	53.4 ± 1.9	52.4 ± 2.0 (−1.0)	<0.01	52.4 ± 1.8 (−1.0)	<0.01
Visceral fat area (cm^2^)	157.7 ± 8.0	152.1 ± 9.0 (−5.6)	<0.01	150.5 ± 8.7 (−7.2)	<0.01
Total body water (kg)	39.5 ± 1.4	38.7 ± 1.4 (−0.8)	<0.05	38.6 ± 1.4 (−0.9)	<0.01
Intracellular water (kg)	24.3 ± 0.9	23.8 ± 0.9	<0.01	23.8 ± 0.9	<0.01
Extracellular water (kg)	15.1 ± 0.5	14.8 ± 0.5	<0.05	14.8 ± 0.5	<0.05

**Table 3:** Various parameters associated with the efficacy of tofogliflozin in univariate analyses.

Comparison	*ρ*	*p*
Baseline HbA1c versus Δ HbA1c (4 w)	0.614	<0.01
Baseline HbA1c versus Δ HbA1c (12 w)	0.630	<0.01
Baseline VFA versus Δ HbA1c (4 w)	−0.169	<0.01
Baseline VFA versus Δ HbA1c (12 w)	−0.019	<0.01
Δ VFA (4 w) versus Δ HbA1c (4 w)	0.371	<0.05
Δ VFA (12 w) versus Δ HbA1c (12 w)	0.177	n.s.
Baseline VFA versus Δ VFA (4 w)	0.993	<0.01
Baseline VFA versus Δ VFA (12 w)	0.002	n.s.

**Table 4:** Determinant factors for the efficacy of tofogliflozin in multivariate analyses.

Explanatory variables	Objective variable	*β*	*p*
(*Model 1*)			
Baseline HbA1c	Δ HbA1c (4 weeks)	0.582	<0.01
Baseline VFA		−0.092	n.s.
Age		−0.178	n.s.

(*Model 2*)			
Baseline HbA1c	Δ HbA1c (4 weeks)	0.647	<0.01
Baseline VFA		−0.076	n.s.
Duration		−0.379	<0.01

(*Model 3*)			
Baseline HbA1c	Δ HbA1c (12 weeks)	0.566	<0.01
Baseline VFA		0.112	n.s.
Age		−0.193	n.s.

(*Model 4*)			
Baseline HbA1c	Δ HbA1c (12 weeks)	0.624	<0.01
Baseline VFA		0.126	n.s.
Duration		−0.307	<0.05
